# Intracellular biomineralization in bacteria

**DOI:** 10.3389/fmicb.2014.00293

**Published:** 2014-06-12

**Authors:** Wei Lin, Karim Benzerara, Damien Faivre, Yongxin Pan

**Affiliations:** ^1^Biogeomagnetism Group, Paleomagnetism and Geochronology Laboratory, Key Laboratory of Earth and Planetary Physics, Institute of Geology and Geophysics, Chinese Academy of SciencesBeijing, China; ^2^France-China Bio-Mineralization and Nano-Structures Laboratory, Chinese Academy of SciencesBeijing, China; ^3^Institut de Minéralogie, de Physique des Matériaux et de Cosmochimie, Université Pierre et Marie Curie, Sorbonne Universités, CNRS UMR 7590, Muséum National d'Histoire Naturelle, IRD UMR 206Paris, France; ^4^Department of Biomaterials, Max Planck Institute of Colloids and InterfacesPotsdam, Germany

**Keywords:** microbial biomineralization, magnetotactic bacteria, magnetosome, magnetotaxis, iron cycling, biosignature, ancient environment

Microorganisms have populated the Earth for billions of years and their activities are important forces shaping our planetary environments through biogeochemical cycles. In particular, microbial biomineralization that selectively take up environmental elements and deposit minerals either intracellularly or extracellularly is of great interest, because these processes play vital roles in the global cycles of numerous elements, such as Fe, Mn, Ca, As, O, S, and P, etc. Biominerals, the products of biomineralization, not only serve as important biosignatures for the search of traces of past life and the reconstruction of ancient environments but also have many important commercial applications.

Recent advances in sequencing technologies, molecular analyses and approaches for assaying protein functions pave the way for rapid progress in microbial biomineralization research. The objective of this research topic is to highlight the latest advances in our understanding of intracellular biomineralization in bacteria, with a focus on the magnetotactic bacteria (MTB), a group of phylogenetically diverse microbes synthesizing magnetic minerals of magnetite (Fe_3_O_4_) and/or greigite (Fe_3_S_4_) magnetosomes in cells. Magnetosomes are generally less than 150 nm in length, covered by lipid bilayer membrane and organized into one or multiple chain structures that act like a compass needle to facilitate the navigation of MTB using the Earth's magnetic field. The uniform nano sizes, superior magnetic properties and perfect chain arrangement suggest a strict genetic control of magnetosome synthesis in MTB cells, which provides an attractive model system for investigating the mechanisms of bacterial intracellular biomineralization. This research topic provides a selection of interesting and challenging topics of research on the diversity, ecology, evolution, genomics, and biochemistry of MTB. The applications of magnetosomes in biotechnology and paleoenvironmental reconstruction are also covered here.

This research topic begins with a review on the relationship between all known magnetosome crystal habits and the phylogenetic affiliations of MTB (Pósfai et al., 2013), which serves as guide to better understand the evolutionary history of magnetosome formation and magnetotaxis in MTB. Following this review, Morillo et al. (2014) report the first cultivation and genomic characterization of a south-seeking *Alphaproteobacteria* magnetotactic coccus *Magnetofaba australis* strain IT-1 from the Southern Hemisphere. The findings of their study provide important clues to the evolution of magnetotactic *Alphaproteobacteria*. Oestreicher et al. (2013) examine the morphological and phylogenetic diversity of MTB in a freshwater lake containing microbialites. Their results raise the interesting question of whether MTB magnetosomes could be preserved in microbialites and serve as robust biomarkers. Kong et al. (2014) investigate the swimming behavior of a rod-shaped magnetotactic *Nitrospirae*, with hundreds bullet-shaped magnetite magnetosomes per cell, under the influence of a magnetic field. The authors have developed the first mathematical model to describe both the magnetotactic motion and orientation of this rod-shaped bacterium.

Three articles deal with the mechanisms of magnetosome biomineralization. Rahn-Lee and Komeili (2013) summarize the recent advances in the molecular mechanisms of magnetosome synthesis and discuss their implications for understanding bacterial intracellular biomineralization in general. Arnoux et al. (2014) focus on those magnetosome-associated proteins containing a *c*-type cytochrome domain. The authors evaluate the evolution of these proteins, and a model is proposed, which offers a different perspective on the evolution of magnetosome formation. Through bioinformatics approaches, Nudelman and Zarivach (2014) perform 3D structural predictions of all known magnetosome-associated proteins in *Magnetospirillum gryphiswaldense* strain MSR-1. Their results propose a comprehensive review of the functional features of biomineralization proteins.

Three papers explore the effects of environmental factors on magnetosome formation and their implications for the ancient environment. Moisescu et al. (2014) review the recent advances in understanding the effects of chemical and physical factors on magnetosome synthesis. They also discuss the potential of magnetosomes as biomarkers for ancient life and the role of MTB in iron cycling. The study by Wang et al. (2013) focuses on the influence of ultraviolet-B radiation on *Magnetospirillum magneticum* strain AMB-1. The authors note that ultraviolet-B radiation could affect both cell growth and magnetite synthesis, and suggest that magnetosomes may have evolutionary benefits for the survival of MTB under extreme environments. A paleo- and rock-magnetic study of marine sediments from the Guadalquivir Basin, Spain by Larrasoaña et al. (2014) reveals dominance of fossil magnetosome chains in sediments. Their results indicate that fossil magnetosomes could provide important paleomagnetic and paleoenvironmental information.

Finally, the articles by Xu et al. (2014) and Yang et al. (2013) focus on the production and application of magnetosomes from *Magnetospirillum gryphiswaldense* strain MSR-1. Xu et al. (2014) report the expression of staphylococcal protein A on the surface of extracted magnetosomes. These functionalized magnetosomes are capable of efficiently detecting pathogenic *Vibrio parahaemolyticus*. Yang et al. (2013) perform experiments with a large-scale culture of *M. gryphiswaldense* and identify the key time point for cell growth as well as magnetosome formation. The high-yield production achieved in their study suggests great opportunities for further applications of magnetite magnetosomes in various commercial fields.

We are very grateful to all the authors for their contributions and to all of the reviewers involved in processing these papers. We hope that this research topic will provide an in-depth exploration of microbial biomineralization and stimulate new investigators and more questions within this fascinating field.

## Conflict of interest statement

The authors declare that the research was conducted in the absence of any commercial or financial relationships that could be construed as a potential conflict of interest.
